# Large area few-layer graphene with scalable preparation from waste biomass for high-performance supercapacitor

**DOI:** 10.1038/s41598-017-15463-w

**Published:** 2017-11-10

**Authors:** Taniya Purkait, Guneet Singh, Mandeep Singh, Dinesh Kumar, Ramendra Sundar Dey

**Affiliations:** Institute of Nano Science and Technology (INST), Mohali, 160062 Punjab India

## Abstract

Carbonaceous materials with high surface area and a sheet-like structure promote fast ion-transport kinetics, making them an ideal choice to be used in supercapacitors. Few-layer graphene (FLG)-like nanosheets with abundance of micro as well as mesopores are achieved via mechanical exfoliation method from an agricultural waste biomass: peanut shell (PS). A well-known elementary method of probe-sonication, for the achievement of FLG sheets from renewable sources, is introduced in this study for the very first time. The Peanut shell-derived FLG (PS-FLG) possesses remarkably high specific surface area (2070 m^2^ g^−1^) with a sufficiently large pore volume of 1.33 cm^3^ g^−1^. For the fabrication of a binder-free supercapacitor, the PS-FLG-based electrodes exhibited a high specific capacity of 186 F g^−1^ without the use of any binder in 1 M H_2_SO_4_ as supporting electrolyte. The highest energy density of 58.125 W h Kg^−1^ and highest power density of 37.5 W Kg^−1^ was achieved by the material. Surprisingly, the working potential increased to 2.5 V in an organic electrolyte leading to an obvious increase in the energy density to 68 W h Kg^−1^. Solid-state-supercapacitor was fabricated with this material for the possible use of low-cost, high energy promising energy storage device.

## Introduction

Graphene, an atomically thin layer of carbon, is an extremely promising two-dimensional (2D) nano material^1^. Since its discovery, initially being regarded only as an ‘academic material,’ graphene emerged as a free-standing 2D planar nano allotrope of elemental carbon, packed in a patterned honeycomb lattice with extremely high carrier mobility suitable for energy applications^2–4^. It has received extensive attention because of its inherent structural flexibility yet substantial mechanical strength, optical transparency and enormous theoretical surface area (2630 m^2^ g^−1^)^1,5^. However, the production of single or few-layer graphene in a simple, efficient, cost-effecitve and scalable pathway is still a massive challaenge in material science.

In the past years, several methods are reported for the synthesis and fabrication of graphene depending upon the applications^6^. Recent research progresses are thus focused on the scalable production of graphene-related materials for commercial applications. However, high scale preparation of graphene with economically valuable and environmentally sustainable methods is still recognized as a challenging issue. Two-dimensional single and/or few-layered graphene has been synthesized by different methods like, (a) chemical vapor deposition (CVD) from decomposition of methane/acetylene/ethylene on metal surface^7,8^, (b) micromechanical exfoliation or scotch tape method from graphite^1,9^, (c) epitaxial growth on electrically insulating surface^10^, (d) chemical method for the production of graphene or reduced graphene oxide (rGO)^11^ and (e) carbonization of biomass/waste materials^12,13^. For the production of graphene without sacrificing the quality of the material; CVD, micromechanical exfoliation, and epitaxial growth have significant roles, but, these methods are not acceptable for economic and large-scale synthesis. However, a chemical process for the production of graphene from exfoliation of graphite oxide can show a route for scalable synthesis, but it involves hazardous and toxic reagents. Moreover, quality of the chemically produced reduced graphene oxide is not suitable for the energy storage device as it suffers from poor electrical conductivity^14^. Recently, researchers are involved in developing a sustainable technique for the large-scale production of carbon nanomaterials including graphene from natural sources (like plant leaves, biochar, waste corn shell, fungus, eggshell, and even human hair)^15–18^.

Bio-waste materials are currently the hotspots because of their abundance, need for recycling and being ample source of carbon. Biomass from plant wastes mainly consists of carbohydrates, fiber, and proteins. Lignin, cellulose, and hemicelluloses are the main components of plant fiber and small amounts of other materials like protein, starches and lipids are also present in there^19,20^. The management of waste biomass has always been a big challenge in smart cities. Hence, bio-waste can be subjected to conversion into carbonaceous materials to achieve economically-worthwhile products for its emergent applications^21^. Biomass and waste materials such as food, agricultural waste, wood waste and other stuff have been utilized for the eco-friendly sources for graphene synthesis^12,13,22,23^. Most of the procedures either used chemical graphitizing agents^12^ or high-temperature graphite furnace^22^ together with activating agents to get graphene of high surface area. Some researchers experimented with multi-step processes (like CVD or plasma enhanced CVD)^24,25^ to grow conductive graphene. These methods are neither scalable nor economical. It is therefore immensely desired to develop a scalable, renewable and cost-effective process for the production of high-quality graphene towards the development of electrode materials for electrical energy storage (EES) devices like supercapacitors and batteries.

Supercapacitors, also known as electrochemical capacitors gained considerable popularity over the last two decades because of their versatile potential in energy storage and delivery. They can store more volumetric or gravimetric energy (energy density) than conventional capacitors; while on the other hand, are able to deliver or accept energy (power density) few orders higher than batteries^26^. Supercapacitors can be fully charged or discharged within seconds and can withstand tens of thousands of charge-discharge cycles without compromising on its energy storage capacity. They are therefore one of the most sought-after EES devices with a broad application range, from hybrid energy vehicles, memory backup and emergency power supply to microelectronic devices like personal digital assistants^27^. Currently, new technologies for the applications of supercapacitors are under challenging research since the performance of the device strongly depends on mechanical strength, surface area, porosity and the production cost of the active electrode materials. Carbonaceous materials including CNTs, activated carbon and their derivatives are setting benchmarks in this flourishing field of supercapacitors, due to their natural biocompatibility, chemical stability, mechanical strength, high conductivity, large surface area and therefore evidently an innate ability to store charges^28–31^. However, most of these materials face a major drawback regarding low energy density (5–8 W h Kg^−1^), low specific capacitance and dynamically poor electronic conductivity^32^. Synthesizing an advanced carbon-based electrode is thus still a significant challenge for the current researchers are dealing with energy storage devices. Graphene stands out to be a better candidate to be employed as an active electrode material for portable supercapacitor devices. State-of-the-art energy-storage devices are thus intrigued with graphene^33^. Single or few layer graphene can offer low-resistant pathway and short ion diffusion channel, which is perpetual solution for high power delivery of supercapacitors^34–36^.

In this work, our objective of using biomass-waste is not only to solve the problem of waste recycling but also to generate value-added materials for renewable energy storage devices like supercapacitors. Peanut shell is a globally-known waste-biomass having no end-use other than being feedstock of animal and building construction materials^37^. According to the Food and Agriculture Organization (FAO) of the United Nations, India is the second largest country after China in peanut production (6.6 million tons per year)^37^. Peanut produces a lot of waste as nutshell all over the world that is approximately six million tons per year^38^. More importantly, peanut shell waste is inexpensive, copious and environmentally benign biological resource. Till now it has been explored only at an research and development scale in the following fields, such as low-cost bio-adsorbent^39^, metal ion removal and wastewater treatment^40–43^, as a catalyst in organic functionalization^44^, fuel gas (H_2_) production^45^, and the hybrid capacitor^38^. Ding *et al*. recently reported both cathode and anode material of a hybrid sodium ion capacitor derived from peanut shell^38^. He *et al*. developed a supercapacitor with mesoporous carbon derived from peanut biomass using ZnCl_2_ activation assisted microwave heating^46^. Activated carbon or mesoporous carbon arising from these methods suffer either from low surface area or high oxygen loading, resulting in a bad conductivity of the materials. It is thus highly anticipated to develop carbon nanomaterials with good conductivity, porous layered structure and high specific surface area, suitable for both fast ion diffusion kinetics and charge storage at the electrochemical double layer for its subsequent applications in supercapacitors. Two-dimensional graphene-like sheets have got the desired morphology, but it has a tendency to restack the layers to get graphite-like structures due to the π-π interaction of the graphene sheets; causing diminished surface area of the resulting materials. Here we have presented a rather common method of mechanical exfoliation in a new perspective by the utilization of no-use peanut shells to obtain value-added few-layer graphene (FLG)-like sheets with an extensively high surface area towards the development of high energy and high-power supercapacitor. Our procedure is highly efficient, environmentally satisfying, economical, scalable and without involving any chemical graphitizing reagent/catalyst.

## Experimental

### Materials and Chemicals

Peanut shells, walnut shells and almond shells were obtained from the local market as waste products. Potassium hydroxide (KOH), potassium bromide (KBr), sulphuric acid (H_2_SO_4_), and isopropanol were purchased from Merck Chemicals, India limited. Tetraethylammonium tetrafluoroborate (Et_4_NBF_4_), ethylene carbonate (EC), N,N-Dimethylformamide (DMF) and Dichloroethane (DCE) were purchased from Sigma-Aldrich. Polyvinyl Alcohol (PVA) was purchased from Alfa-Aesar. All other reagents used in this study were of pure analytical grade and were used without any further purification. All aqueous solution was prepared using mili-Q water.

### Characterization techniques

Fourier transform Infrared (FTIR) spectroscopy was carried out on an Agilent technology Cary 600 series FTIR instrument at room temperature. For FTIR analysis, PS-FLG was mixed with KBr and then finely ground to make a pellet. Raman Spectroscopy was performed on a WITEC Focus Innovations Alpha-300 Raman confocal microscope at a laser wavelength of 532 nm. X-ray Diffraction (XRD) spectroscopic study was carried out on a Bruker D8 Advances instrument using Cu-Κα (λ = 1.5406 Å) radiation in the 2θ range from 5° to 80° with an acceleration voltage of 40 KV. Nitrogen adsorption-desorption analysis was done at 77 K on an Autosorb iQ2 instrumental setup to examine the surface area by Brunauer Emmett Teller (BET) method. The pore size distribution was computed by the nonlocal density functional theory (NLDFT) technique. The samples were degassed at 300 °C for more than 12 h under vacuum conditions. The surface morphology and the elemental composition of the synthesized material were investigated using Scanning Electron Microscopy (SEM Jeol JSMIT300) equipped with a Bruker XFlash 6130 Energy Dispersive X-ray Spectroscopy (EDS) at each stage of synthesis. Probe sonication was performed on a QSonica (Part no.-Q700, USA) ultrasonicator using a replaceable microtip of the diameter of 1/16″ (1.6 mm) at an amplitude of 112 µm (35%). Atomic force microscopy (AFM, Bruker Multimode 8) was used to investigate the surface topologies of the synthesized active material (PS-FLG). Transmission Electron Microscopy (TEM) studies were carried out on a JEM2100 instrument, equipped with digital micrograph software for investigating Selected Area Electron Diffraction (SAED) pattern of the graphene sheets. All electrochemical experiments, like, cyclic voltammetry, galvanostatic charge-discharge and electrochemical impedance spectroscopy (EIS) were performed in 1 M H_2_SO_4_ solution aqueous electrolyte and 1 M Et_4_NBF_4_ (in 1:1 EC: DCE) organic electrolyte using a CHI 760E electrochemical workstation. Glassy carbon (GC) disc electrode was used to load the active material and studied for their electrochemical behavior. A three-electrode cell setup with a GC working (0.07 cm^2^), a platinum wire auxiliary and Ag/AgCl (3 M KCl) reference electrodes were used in the voltammetric and chronopotentiometric measurements. A similar setup was organized for electrochemical testing in the organic electrolyte system except for Ag/Ag^+^, which was taken as the reference electrode. Sigma DC regulated power supply (0–30 V, 0–5 A) was used for charging the solid-state devices to constant potential.

### Synthesis of graphene from waste peanut shell

**Figure 1:**
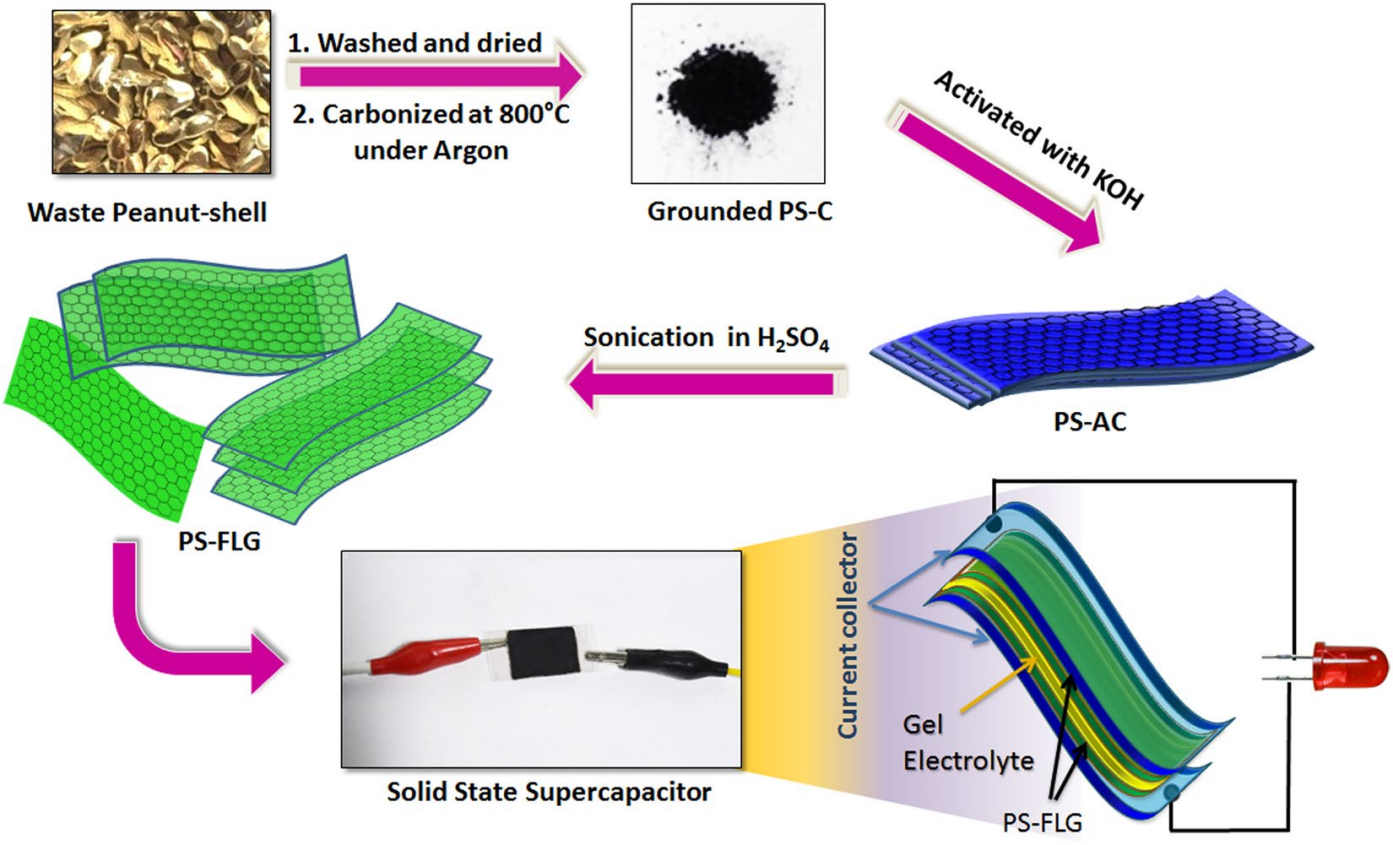
Schematic representation for the synthesis of PS-FLG active material and its subsequent integration into a solid-state device.

Peanut shell-derived few layer graphene (PS-FLG) was prepared by a simple activation (by KOH) followed by mechanical exfoliation method. Peanut shells wastes were collected from the local market as waste products and purified by the following method. The waste material was copiously washed with distilled water to remove dust and other interfering particles and dried in sunlight for a few days. Then it was dried in a vacuum oven at 80 °C for overnight to remove any moisture content. These shells were crushed into a fine powder which was stored in the completely dried atmosphere. For the synthesis of carbon nanomaterials, the peanut shell precursor (PS-P) powder (5.0 g) was pyrolyzed in a tubular furnace at 800 °C for two hours under an argon atmosphere at a heating rate of 3 °C min^−1^. The resulting carbonized product of peanut shell (PS-C) was washed thoroughly with isopropanol to remove any unwanted organic deposition. Then the carbon powder was mixed with a well-known porogen KOH (w/w 1:3) and mixed it through mortar-pestle to get a homogeneous mixture. This homogeneous mixture was then heated in a tubular furnace under an argon atmosphere at 800 °C for another two hours to generate the required porosity and functionality to get peanut shell derived activated carbon materials (PS-AC). The activated sample was then washed with 1:1 HCl solution followed by distilled water until the pH reached neutrality and dried for overnight. The exfoliation of the as-prepared PS-AC sample was performed in 10% H_2_SO_4_ aqueous solution through probe sonication for 1 hour with a power of 15 W and a pulse on-and-off time of 5 seconds. The tip was dipped well inside the solution for better sonication, but a distance of at least 2 cm was maintained from touching the bottom of the beaker containing the solution. The desired temperature was maintained throughout by using an icebath and by carrying out the experiment in 5 seconds pulses separated by 5 seconds cooling period. Finally, the product was repeatedly washed with isopropanol and deionized water to remove any impurity if present in the sample. The washed samples were centrifuged and dried at 80 °C for overnight. The final product is named as peanut shell derived exfoliated few-layered graphene (PS-FLG). The weight of the PS-FLG material synthesized from peanut shell waste was calculated to be 1.125 g and the corresponding yield was found to be 22.5 wt % with respect to PS-P material. Figure 1 shows the schematic pathway used for the synthesis of PS-FLG from precursor PS-P. Walnut shell-derived carbon (WS-C) and almond shell-derived carbon (AS-C) were prepared from waste walnut shells and almond shells, respectively, for comparison with the capacitive behavior of PS derived material. Same procedures as described above were followed maintaining similar experimental conditions.

### Fabrication of supercapacitor electrode

A cleaned Glassy carbon (GC) electrode was used as a conductive current collector for evaluation of the electrochemical performance of the as-developed materials in a three-electrode setup. Before use, the GC electrode was polished with fine alumina powder and repeatedly washed with DI water followed by sonication in water. The active materials (PS-C, PS-AC, and PS-FLG) were dispersed in1:1 isopropanol-water mixture and sonicated for 15 min to get a homogeneous dispersion. The solution was then drop cast on the pre-cleaned GC electrode and dried overnight at room temperature in vacuum. The concentration of all the active materials loaded on GC electrode was 1 mg cm^−2^. The electrode was subjected to all electrochemical measurements performed in a three-electrode system. Cyclic voltammetry (CV), galvanostatic charging-discharge (GCD) and electrochemical impedance spectroscopy (EIS) were carried out using CHI 760E electrochemical workstation. All the electrochemical measurements were performed between a potential window of −0.4 to 1.1 V in 1 M H_2_SO_4_ aqueous electrolyte and −1.3 to 1.2 V in 1 M Et_4_N-BF_4_ organic electrolyte.

The solid-state supercapacitor was made with PS-FLG powder coated on ITO sheets and assembled together with gel electrolyte. Briefly, the PS-FLG powder material was first dispersed in DMF solution, and dense ink was made out of it. The ink was then coated onto thin ITO sheet using doctors blade and dried under vacuum overnight. After drying, two of such PS-FLG coated ITO sheets were symmetrically sandwich together with PVA-H_2_SO_4_ as an ionogel electrolyte to construct an all-solid-state supercapacitor. The ionogel electrolyte was prepared by adding 1 g of polyvinyl alcohol (PVA) in 10 ml water and stirred at 90 °C until the solution becomes clear. A stoichiometric amount of H_2_SO_4_ solution (1 M) was then added to the mixture, and the stirring was continued for another 1 h. The device was allowed to dry at room temperature for 24 h before testing its performance.

## Results and Discussions

It is crucial to design advanced electrode materials to meet the rising and urgent demand for high-performance supercapacitors. An inexpensive carbon-based electrode would not only provide a cost advantage but would also maximize the device energy density. Peanut shell-derived few-layer graphene (PS-FLG) was so chosen as the active material for supercapacitor. First, peanut shell precursor was carbonized followed by activation with KOH. Potassium reacts violently to rip apart the layers between carbons. To get the highly dispersed PS-FLG, probe ultrasonication was preferred to accelerate the aqueous exfoliation of PS-AC in H_2_SO_4_ because a definite power delivery into the aqueous system can be ensured in this process; entirely different from the variable bath ultrasonication^47^. Probe sonication was performed on a QSonica (Part no.- Q700, USA) ultrasonicator using a replaceable microtip of a diameter of 1/16″ (1.6 mm) at an amplitude of 112 µm for 1 h with a pulse on-and-off time of 5 seconds.

To check the dispersion of the as derived PS-FLG material in different solutions, several aqueous, as well as organic solvents have been selected. Figure S1 shows the digital photographs of PS-FLG dispersed in various solvents like water, isopropanol, dimethylformamide, dimethyl sulfoxide and carbon tetrachloride. The PS-FLG sample exhibits high dispersibility in polar (aqueous) as well as non-polar (organic) solvents. Fourier-Transform Infra-Red (FT-IR) analysis was conducted to establish the functional group present in PS-FLG material. As can be observed in Fig. S2, an array of both polar and non-polar functional groups is present in PS-FLG. The absorption peak at 3429 cm^−1^ can be assigned for the -OH stretching vibrations of the hydroxylic group and chemisorbed water^48^. The skeletal ring vibration of graphene-like sheets can be observed at 1631 cm^−1^ ^49,50^. The peak at 3160 cm^−1^ stands for C=C-H stretching mode^51^. Medium broad vibration observed at 2786 cm^−1^ is ascribed to the overtones of O-H bending mode which arise from proton tunneling and Fermi resonance interactions^52^. The peaks at 1397 cm^−1^ and 1464 cm^−1^ appears because of the-CH_2_ and -CH symmetric bending modes^53^. Presence of both polar and non-polar groups in its structure, makes PS-FLG disperse both in aqueous or organic solvents.

**Figure 2:**
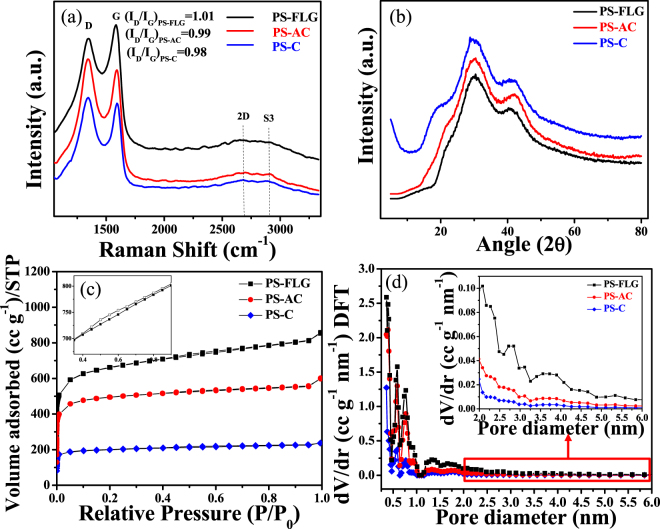
(**a**) Raman spectrum; (**b**) XRD patterns; (**c**) Comparison of N_2_ sorption analysis isotherm (magnification in inset showing a type-IV isotherm for PS-FLG) and (**d**) Pore size distribution plot by NLDFT method of PS-derived materials at different stage of synthesis;(inset shows achievement of mesopores for PS-FLG).

In order to investigate the structural transformations of the as-synthesized PS derived carbon materials, Raman spectral analysis was carried out. Figure 2a records all the Raman peaks corresponding to the PS-derived carbon structures at various stages of synthesis. Raman spectra of graphene-like materials are regularly characterized by two featured bands, D-band & G-band^54^. The D-band arise due to the breathing mode of k-point phonons of A_1g_ symmetry and G-band is assigned to the first order scattering of E_2g_ phonon of sp^2^ carbon atoms^21,55^. As can be observed in Fig. 2a there is a gradual red shift in the D-peak from PS-C (1351 cm^−1^) to PS-AC (1344 cm^−1^) to PS-FLG (1342 cm^−1^); it can be assigned to the gradual attainment of the more ordered structure of the PS-derived material at each stage. The G-band appears because of degenerate in-plane E_2g_ optical mode at the center of the Brillouin zone of graphitic carbon^56^. PS-derived carbons show a red-shift also in their G-band from PS-C (1593 cm^−1^) to PS-AC (1590 cm^−1^) to PS-FLG (1588 cm^−1^); gradually reaching for the more graphitic character. A characteristic overtone peak (2D band) at 2680 cm^−1^and (S3 band) at 2909 cm^−1^ appeared corresponding to PS-FLG, indicating graphitization of the materials^57^. The degree of crystallinity is directly proportional to the intensity ratio of D & G-peaks. As calculated from the obtained data, I_G_/I_D_ for PS-FLG is 1.01, gradually increasing from 0.99 for PS-AC & 0.98 for PS-C; suggesting the increase in crystalline nature of the material^12^.

Wide angle X-ray powder diffraction analysis of the PS-FLG sample was conducted under monochromatized Cu-K_α_ to study the crystallinity and phase structure. As shown in Fig. 2b, the prominent diffraction peak at 2θ = 30.1° originates because of the characteristic reflection from the (002) graphitic plane. This peak is much sharper than the small peak at 2θ = 43.5° assigned to the (100) plane of distorted graphitic sheets, confirming the high degree of crystallinity of the exfoliated sample^58^. Figure S3 gives a comparative view of the XRD patterns of WS-C and AS-C. It shows the presence of some turbostratic disordered carbon phase along with the characteristic diffraction peaks for carbonaceous materials, at 2θ = 23.9° corresponding to the (002) plane and 2θ = 43.1° for the (100) plane in both AS-C and WS-C^59^.

Specially designed layered carbon nanomaterials with a good balance between mesoporosity and microporosity is highly desirable to achieve high energy/power density simultaneously^60^. Materials with high surface-to-volume ratio and abundance of mesopores promote sufficient charge storage (high energy density) and fast charge transfer kinetics (high power density) respectively which are very significant for applications in advanced energy storage systems. The N_2_ adsorption-desorption isotherm of PS-FLG shows a mixed type I and type IV curve as observed in Fig. 2c; typical of a material with both microporosity and mesoporosity. A magnified image, of the curve corresponding to PS-FLG shown in the inset of Fig. 2c, shows a typical type IV curve with an H_2_ type hysteresis loop, inherent of a mesoporous material, in the relative pressure (P/P_0_) range of 0.4–0.7^12,60^. However, this type of hysteresis loop is absent in the case of PS-C and PS-AC (Fig. 2c), suggesting the presence of only micropores in them. Only micropores are not suitable for either charge storage or propagation^12^. A steep increase in N_2_ uptake at lower relative pressure also suggests the presence of micropores in PS-FLG^61^. Mechanical exfoliation not only separates the graphene sheets, but it is also involved in increasing the porosity, suitable for supercapacitors. Pore size distribution was computed using the adsorption branch of the isotherm by the NLDFT method, showing an average pore size of 1.3 nm in Fig. 2d. Magnified view of the NLDFT pore size distribution plot of PS-FLG (Fig. 2d, inset) reveals achievement of mesopores (2.1–5.4 nm) during exfoliation. Microporous substances with an enhanced surface area is ideal for elevated capacitance and power density in aqueous electrolyte because of high conductivity and smaller ionic size^62^. But to explore its potential in the organic electrolyte, it is necessary to have a reasonable mesopore volume and their good interconnectivity for the electrolyte ions to effectively realize all the available surface area of the carbon framework^63,64^. The specific surface area of the PS-FLG is measured to be 2070 m^2^ g^−1^, whereas, the surface area of PS-C and PS-AC is estimated to be 645 and 1554 m^2^ g^−1^, respectively. This high surface area acknowledged the superiority of the methods used to produce PS-FLG material compared to other methods reported recently for producing graphene-like carbon from biomass^13,22,23,65,66^ (Table 1). Such a high specific surface area (2070 m^2^ g^−1^) with high pore volume (1.33 cm^3^ g^−1^) can afford more active sites during the charge-discharge process and thus makes PS-FLG an attractive material for energy storage devices. WS-C and AS-C were also subjected to BET surface area analysis and the specific surface area of the WS-C and AS-C are measured to be 363 and 403 m^2^ g^−1^, respectively. Figure S4a gives the comparative N_2_ adsorption-desorption isotherm plot of PS-FLG with the other two nutshells-derived carbon. Magnified view of WS-C and AS-C are shown in Figure S4b, which represents that both the materials possess type IV curve with an H2 type hysteresis loop, typically observed for mesoporous materials. Though the average pore size of both the material is around 2 nm, the presence of pores with larger diameters (Figure S4c) are also observed, confirming the presence of mesopores in them. Although the materials have mesoporous architecture, their not-so-high specific surface area along with low pore volume (0.047 cm^3^ g^−1^ for WS-C and 0.035 cm^3^ g^−1^ for AS-C) is responsible for their lower capacitive properties than PS-FLG. Table S1 summarizes all the surface morphological analysis data obtained from the peanut, walnut and almond shell-derived carbon material.

**Table 1:** Comparison of different graphene-based materials synthesized from waste biomass and their applications.

Waste source	Scientific name	Synthetic route		Surface area (m^2^ g^−1^)	Application	References
		Activating agent	Graphitization process			
Coconut shell	*Cocos Nucifera*	ZnCl_2_	FeCl_3_	1874	Supercapacitor	12
Soybean shell	*Glycine Max*	KOH	Thermal treatment with NH_3_ injection for N-doped graphene	1152	Oxygen reduction reaction	13
Wheat straw	*Triticum sp*.	KOH	Thermal treatment in graphite furnace	35.5	Li-ion battery	22
Tea tree plant	*Melaleuca Alternifolia*	Plasma enhanced chemical vapor deposition		—	Hydrophobic coating	23
Auricularia + GO	*Auricularia auricula-judae*	KOH	Hydrothermal carbonization with GO	1723	Supercapacitor	65
Waste paper (Co decorated porous graphene)	—	—	Graphitization & Co(acac)_2_ + 1,10 phenanthroline	542	Oxygen reduction reaction	66
**Peanut shell**	***Arachis Hypogaea***	**KOH**	—	**2070**	**Supercapacitor**	**This work**

**Figure 3:**
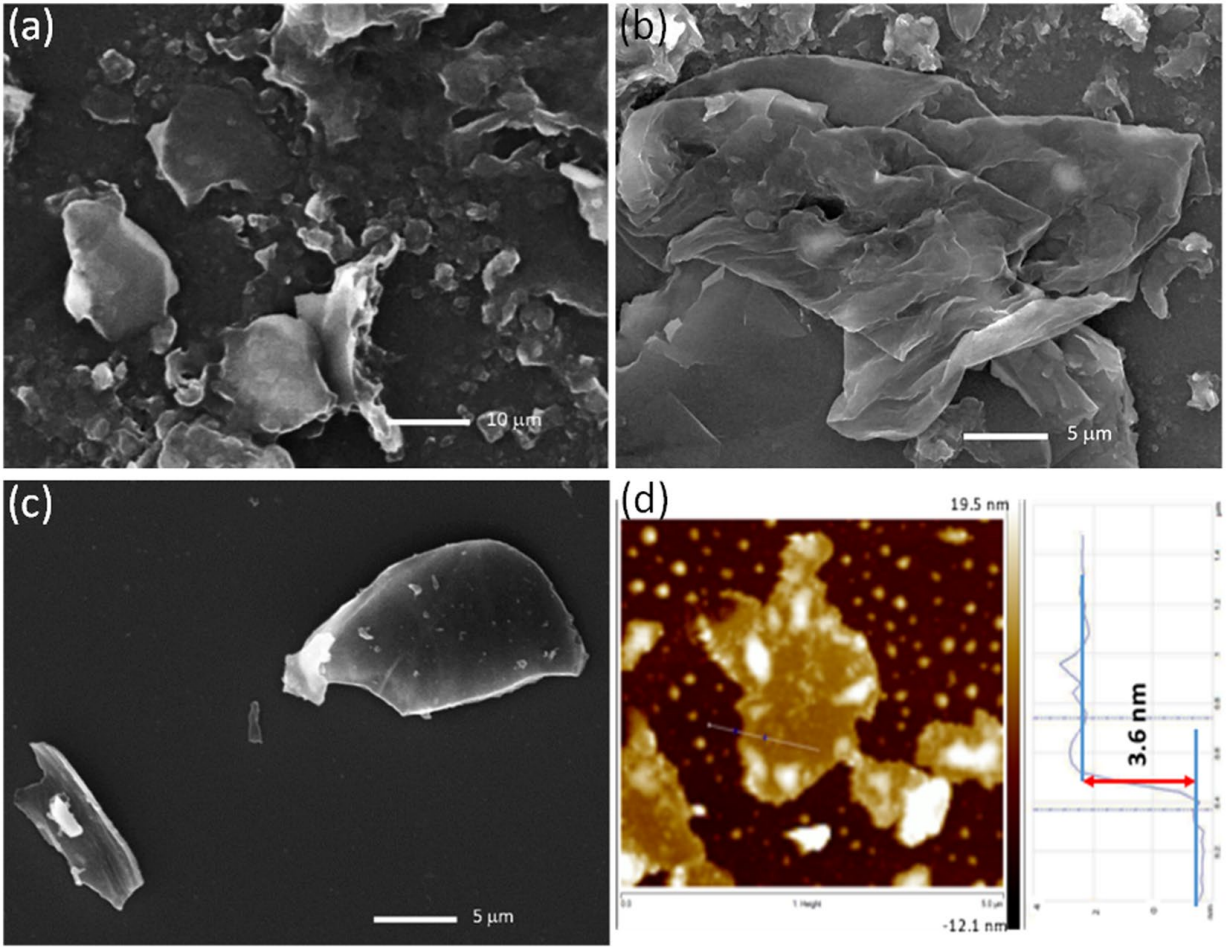
Microscopic surface analysis of exfoliated PSC (PS-FLG): (**a**), (**b**) and (**c**) SEM images at different resolutions. Scale bars: 10 µm and 5 µm. (**d**) Tapping-mode AFM images representing 2D mapping and height profile.

To investigate the microstructures, surface morphology and topology of the as-synthesized materials, SEM and AFM investigations were performed. The morphology of PS-FLG can be attributed to our synthesis strategy which is tailored accordingly to take maximum structural advantage of the peanut shell. SEM images in Fig. 3a–c reveals that the so formed PS-FLG contains a broad distribution of sheet-like structure. Figure S5a shows the distinctive SEM micrograph of the pre-carbonized PS, where the naturally abundant lignocellulosic microfibril networks quantify the rough surface morphology, a characteristic feature of any biomass. Figure S5b and c show the SEM images of the carbonized sample PS-C, and KOH-activated sample PS-AC, respectively. As observed, on activation with KOH at 800 °C the samples transformed to sheet-like morphology. Further, as seen in Fig. 3a–c, on exfoliation the otherwise stacked sheet-like structure was transformed to few layers graphene-like morphology with nearly transparent appearance. SEM images of walnut and almond waste shell based carbon were also captured. As can be observed in Figure S6, both WS-C and AS-C have a porous surface with profoundly broken sheet structures forming agglomerates unlike the continuous few-layer graphene-like nanosheet morphology, observed for PS-FLG in Fig. 3.

EDS was carried out to find out the elemental composition of the as developed PS-FLG, as observed in Fig. S7. The abundance of carbon is clearly seen with almost 94 wt.% of it with the minimal occurrence of oxygen (only about 6 wt.%) confirming the effective carbonization of the active materials. Atomic force microscopic (AFM) measurements were conducted to study the top-view images and cross-sectional thickness of the FLG material. The average cross-sectional thickness of the graphene sheets is found to be around 3.2 nm. Figure 3d reveals the existence of 6–8 layers of graphene sheets, considering the thickness of the monolayer graphene is 0.33 nm^67^. A detailed three-dimensional mapping of the PS-FLG nanosheets is presented in Fig. S8.

**Figure 4:**
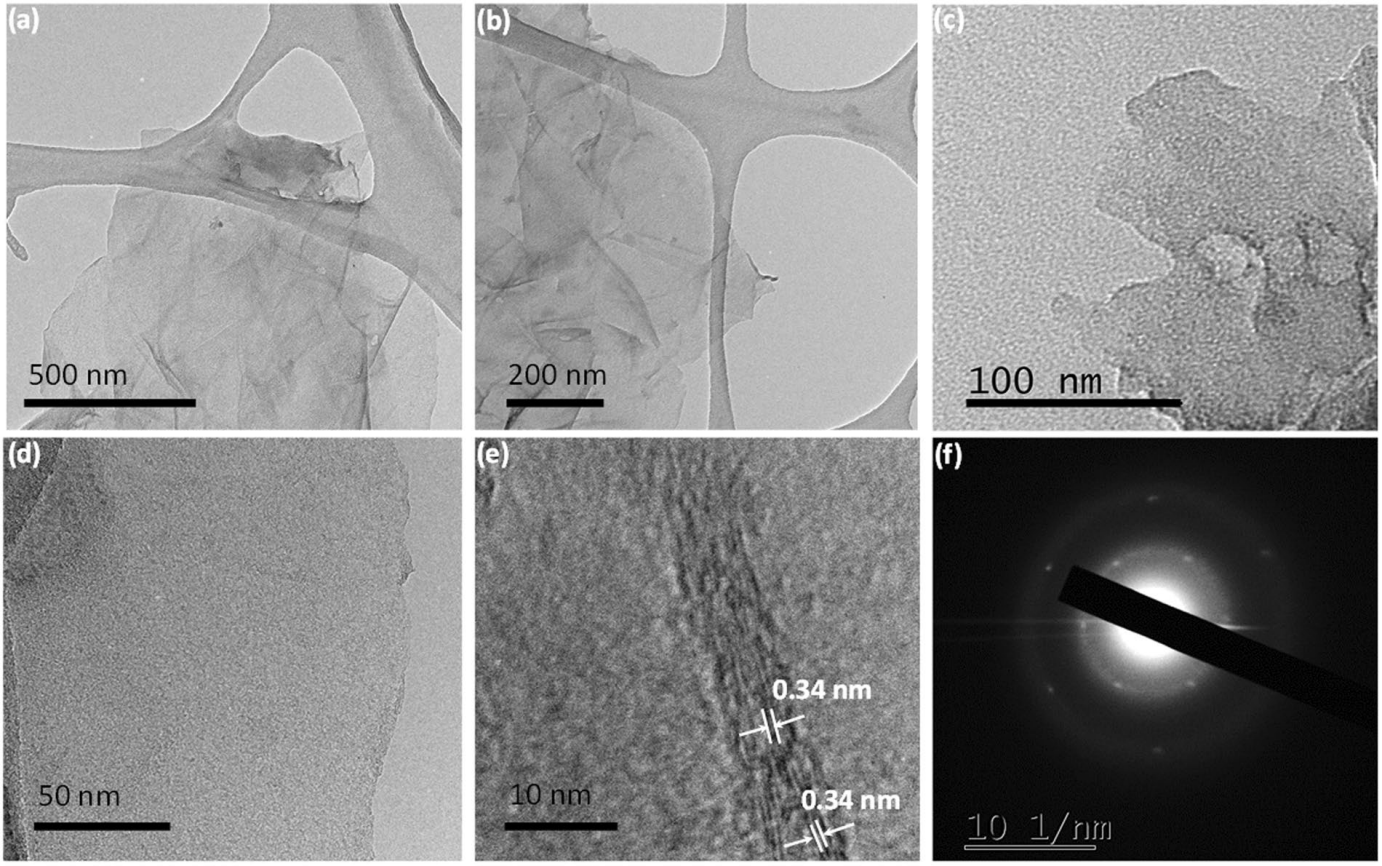
(**a**–**c**) TEM images of PS-FLG at different magnifications; (**d**) and (**e**) HRTEM images showing crystallite size of about 4.2 nm; (**f**) SAED pattern of PS-FLG clearly showing hexagonal graphene like lattice.

TEM measurement was performed to confirm the morphology of the few-layered graphene materials further. As can be seen in Fig. 4a–c, transparent few-layer graphene-like nanosheets are to be clearly observed at various magnifications on a holey carbon grid. High-resolution TEM (HRTEM) images of selected regions are presented in Fig. 4d and e revealing distorted nanosheets consisting about 5–6 layers of graphene nanosheets. Further, HRTEM confirms the interlayer spacing of ~0.34 nm (Fig. 4e), which corresponds to the (002) plane of few-layered graphene nanosheets. Selected area electron diffraction (SAED) analysis reveals the presence of a hexagonal lattice of crystalline graphene-like sheets with long-range order^62^. As can be observed from the diffraction pattern in Fig. 4f, there are six well-defined diffraction spots corresponding to a graphene-like lattice, indicating the crystalline nature of the sheets^68,69^. The active crystal planes of the nanosheets of PS-FLG are calculated to be (002) and (100) planes of the reciprocal lattice, which is in line with the data obtained from XRD measurements (Fig. 2b).

### Electrochemical Analysis

**Figure 5:**
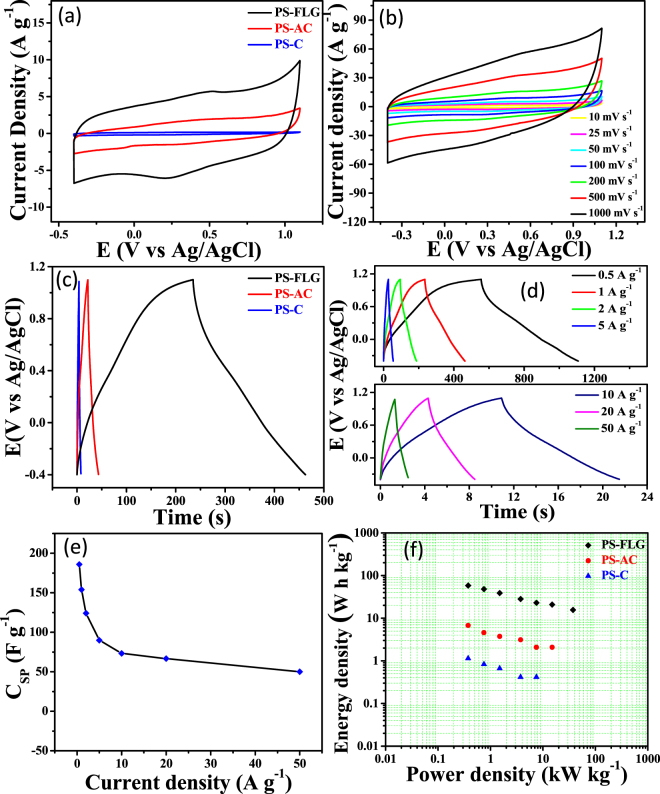
Electrochemical capacitive behavior of different PS based electrodes in 1 M H_2_SO_4_: (**a**) CV at a scan-rate of 100 mV s^−1^ at different stages of synthesis; (**b**) typical CV response of PS-FLG at various scan rates (10–1000 A g^−1^); (**c**) GCD curves comparing at 1 A g^−1^; (**d**) GCD of PS-FLG at different current densities from 0.5–50 A g^−1^; **(e**) change in specific capacitance of PS-FLG as a function of current density; (**f**) comparative Ragone plot.

Electrochemical performances were measured to evaluate the potential applications of the PS-derived few layer graphene-like nanosheets as an active electrode material in aqueous as well as organic electrolyte using three electrode setup. Cyclic voltammetric curves obtained in 1 M H_2_SO_4_ at different stages of synthesis show the gradual increase in specific capacitance (C_SP_) from the carbonized sample, PS-C; to the activated sample, PS-AC; and to the exfoliated sample, PS-FLG as exhibited in Fig. 5a. The increase in the C_SP_ of PS-FLG with respect to PS-C and PS-AC is due to the increased specific surface area as well as the better distribution of pores of slightly larger diameter (as shown in Fig. 2d, inset); which favours the charge storage capacity of the exfoliated material. It explains the phenomenon of better ion transport of the PS-FLG materials; which further establishes the need for exfoliation to attain well-separated nanosheets. A distinctive capacitive behavior with quasi-rectangular CV curve was maintained throughout a good range of scan rates from 10 mV s^−1^ to 1000 mV s^−1^ for PS-FLG as shown in Fig. 5b, implying quick dynamics of high power behavior of the PS-FLG material. The presence of a bump in CV at slow scan rate indicates a little contribution of redox species to the electrochemical capacitance of the PS-FLG materials. The closely rectangular shape of the CV was maintained above the scan rate of 100 mV s^−1^, which may be attributed to the optimum amount of combined micropore and mesopore volume as well as good electrical conductivity^63^. The capacitive potential of the PS-FLG based electrode was further investigated by galvanostatic charge-discharge (GCD) experiments. An expected increase in discharge time was observed in the case of PS-FLG from PS-AC or PS-C at a current density of 1 A g^−1^ as shown in Fig. 5c.

The highest C_SP_ of 186 F g^−1^ was obtained at a current density of 0.5 A g^−1^ for PS-FLG as can be calculated from Fig. 5d. The superior performance of PS-FLG can be ascribed to its porous sheets like structure and the high BET surface area (2070 m^2^ g^−1^) which is crucial for better penetration of electrolyte ions and fast charge propagation and storage. Exfoliation promotes a few atomic layered thick graphene sheets, increasing its electrical conductivity dramatically and thus PS-FLG shows a huge increment in capacitive performance as an active electrode material for supercapacitor. The C_SP_ (F g^−1^) of PS-FLG-based electrode material were calculated at various current densities from 0.5 A g^−1^ to 50 A g^−1^ (Fig. 5d). Rate capability is one of the determining parameters that are essential for practical applications of the active electrode material. Here as can be seen, even at a higher current density (50 A g^−1^), the typical quasi-rectangular shape of the GCD curve is maintained showing appreciable charge-discharge reversibility. As with enhanced current density, limited charge diffusion tends to reduce the specific capacity; Fig. 5e demonstrates how excellently PS-FLG can retain C_SP_, even with increasing current density.

For the fabrication of highly advanced supercapacitor for practical use, energy density must be improved without sacrificing the power supply. Enhanced C_SP_ with a wide operating potential window is appropriate for increasing the energy density of the supercapacitor material. From the Ragone plot (Fig. 5f), it can be observed that PS-FLG exhibits highest energy density of 58.13 W h Kg^−1^ against a reasonably good power density of 375 W Kg^−1^ in a large potential window of 1.5 V (−0.4 V–1.1 V). This is maintained at 15.63 W h Kg^−1^ even at the highest power density of 37.5 KW Kg^−1^ in an aqueous electrolyte of 1 M H_2_SO_4_. The PS-AC and PS-C show highest energy densities at 6.8 W h Kg^−1,^ and 1.2 W h Kg^−1^ with highest power densities attained at 15 W Kg^−1^ and 7.5 W Kg^−1^, respectively (Fig. 5f).

**Figure 6:**
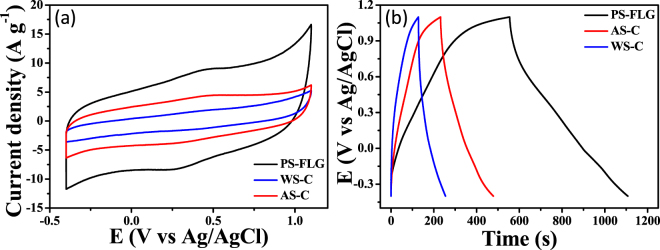
Comparison of electrochemical capacitive behavior of different nutshell-based electrodes in 1 M H_2_SO_4_: (**a**) CV at a scan-rate of 100 mV s^−1^; (**b**) GCD curves at a current density of 0.5 A g^−1^.

Electrochemical responses of the walnut and almond shells-derived carbon (WS-C or AS-C) were also studied as potential materials for different carbon sources for comparison. Cyclic voltammetry (Fig. 6a) at a scan rate of 100 mV s^−1^ and galvanostatic charge-discharge study at 0.5 A g^−1^ (Fig. 6b) was conducted for WS-C and AS-C to compare the capacitive behavior with PS-FLG material. As can be observed in Fig. 6b, PS-FLG shows a huge C_SP_ of 186 F g^−1^, much more (about 4.5 times and 2.5 times higher) than that indicated by WS-C (42 F g^−1^) or AS-C (78 F g^−1^) at a current density of 0.5 A g^−1^. Detailed electrochemical performance of WS-C and AS-C are shown in Fig. S9. Fig. S9a and b gives the cyclic voltammetric responses at various scan rates while Fig. S9c and d represent the galvanostatic charge- discharge profile at different scan- rates of WS-C and AS-C based electrodes. As can be seen in Fig. S9c and d, both WS-C and AS-C are stable only up to a current density of 10 A g^−1^, much lower than PS-FLG which can retain their rate capability even at a much higher discharge current of 50 A g^−1^.

**Figure 7:**
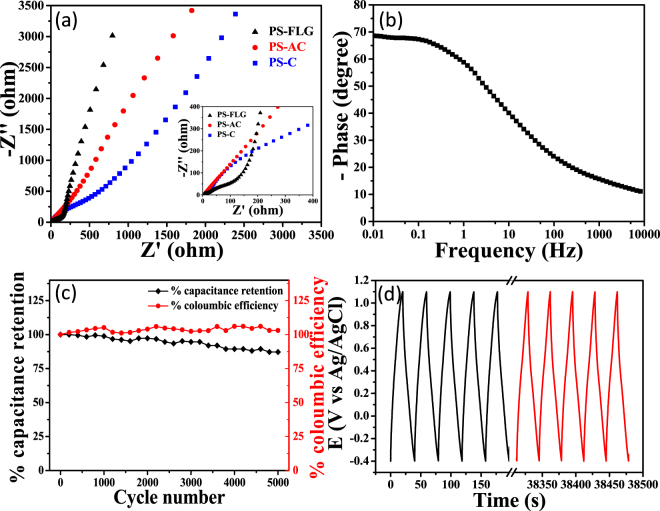
Electrochemical Impedance Spectroscopy: (**a**) Comparative Nyquist plot at an AC amplitude of 5 mV. **(b**) Bode plot of PS-FLG at a range of 0.01 Hz to 10 kHz. (**c**) Charge-discharge cycling test, retention of specific capacitance and coloumbic efficiency with number of cycles. (**d**) Cycling stability of PS-FLG showing initial & final 5 cycles of GCD at a current density of 5 A g^−1^.

Electron impedance spectroscopy (EIS) was further done at the open circuit potential (OCP) to ensure that the as developed few-layer graphene-like nanosheets are well equipped to promote fast charge-discharge as well as efficient charge storage^12^. As demonstrated in Fig. 7a, at lower frequency region, the imaginary part increases more sharply for PS-FLG, than PS-C & PS-AC, indicating diffusion limited electron transfer characteristic of ideal capacitive behavior of the material. A closer view of the mid-frequency region reveals that the line due to Warburg resistance cuts the real axis at the smallest angle for PS-FLG as compared to other PS-based electrode materials. It indicates that the ion diffusion pathways for electrolyte ions to the pores of the electrode surface is shortest for PS-FLG^12^. A magnified image (inset, Fig. 7a) in the higher frequency region shows the real axis intercept provides equivalent series resistance (ESR, R_s_) along with the semicircular impedance loop whose diameter gives the charge transfer resistance (R_ct_). The R_s_ value for the PS-FLG material is found to be 6.1 Ω, whereas, it was 6.8 Ω and 25.4 Ω, for PS-AC and PS-C, respectively. The R_s_ value clearly tells the PS-FLG material has high conductivity and low internal resistance. The nanosheet-like structure allows for fast ion-transfer kinetics between the electrode surface and electrolyte making it most suitable for use as an active material. Figure 7b is the Bode phase plot, which demonstrates that in the low-frequency region when the phase shift approaches −70°, the material performs more like capacitors attends with the diffusion process^70,71^. The phase angle reaches −45° at the capacitor response frequency of 5.6 Hz, which is much higher than activated carbon (0.15 Hz)^72^.

Galvanostatic charge-discharge cycling stability is one of the critical parameters to look out for in commercially available supercapacitors. The active material must endure fast charging-discharging at higher current densities and yet maintain its inherent property even after large number of GCD cycles. Figure 7c exhibits a promising 87% C_SP_ retention even after 5000 GCD cycles at a sufficiently higher current density (10 A g^−1^). Fig. 7d shows the initial and final five GCD cycles having almost similar triangular pattern retained as is expected for good charge propagation of electrolyte ions into PS-FLG electrodes. CV plots before and after GCD cycling test can be observed in Figure S10, showing a slight decrease in the area under the curve but the characteristic CV shape is retained. This confirms the cycling stability of the active material. Figure 7c also shows retaining of coloumbic or faradaic efficiency around 100% indicating an absence of any side reactions or faradaic processes were taking place during GCD cycling test.

**Figure 8:**
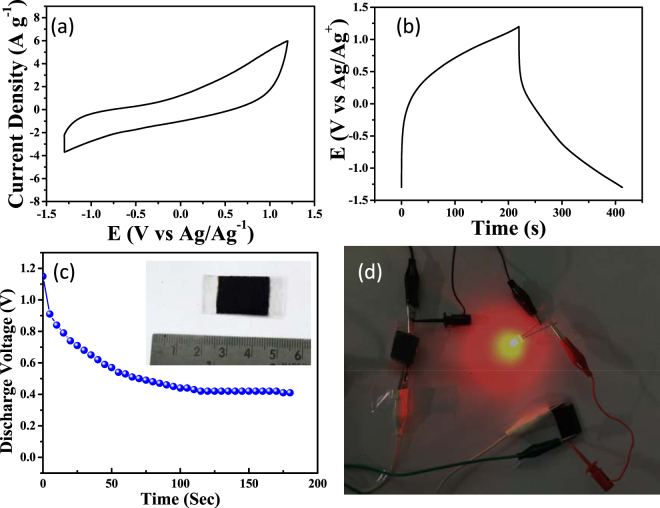
Electrochemical performance of PS-FLG in 1 M Et_4_NBF_4_ in 1:1 EC: DCE; (**a**) CV response at a scan rate of 100 mV s^−1^; (**b**) GCD test at a current density of 1 A g^−1^. (**c**) Plot of discharge voltage of a SSD recorded with time. Inset is the optical image of a SSD device. (**d**) Lighting a red LED by two devices connected in series.

The capacitive behavior of PS-FLG material was tested in an organic electrolyte. Briefly, the CV and charge-discharge test were performed in 1 M Et_4_NBF_4_ in 1:1 mixture of EC: DCE solvent with a three-electrode setup. Figure 8a exhibits the cyclic voltammetric response of the PS-FLG active electrode material in the organic electrolyte system. A high C_SP_ of 78.3 F g^−1^ at a current density of 1 A g^−1^ during galvanostatic charge-discharge study was achieved (Fig. 8b). Introducing organic electrolyte allows an increment of the potential window to 2.5 V, thereby increasing the energy density to 68 W h Kg^−1^.

A comparative study of carbonaceous materials obtained from various waste biomass sources and their capacitive performances are presented in Table 2. A supercapacitor needs to have sufficiently high energy density without sacrificing on its instant power delivery, to be employed for commercialization. It is mostly observed that any material, which is stable even on a more extensive potential range, subsequently has higher energy storage capacity. Mostly reported supercapacitors based on carbon nanostructures are limited to function within a potential range of 1 V in an aqueous electrolyte^12,21,73–77^. However, our as developed PS-FLG material is shown to be functional in a much higher potential window of 1.5 V. Another key factor lies in the attainment of morphologies suitable for maximum charge storage. The predominant product morphology for the carbonaceous materials from the majority of the waste sources is found to be mostly activated or porous carbon. Only activated carbons from dried corn grains or sugarcane bagasse has got limited energy densities at a range of 10–30 W h Kg^−1^ ^75,77^. On the other hand, nano or micro porous carbons from various sources like wood sawdust, dead neem leaves or waste coffee beans exhibit slightly better energy densities in the range of 8–55 W h Kg^−1^ (power densities ranging from 5–12 KW Kg^−1^) mostly because of the presence of well-distributed pores^48,57,78^. This establishes the need for a combination of both a layered structure with well-distributed pores and sufficiently high surface area for the achievement of higher energy density. PS-FLG possesses all these qualities along with an enhanced potential window. It shows almost 6 times higher energy density (58.25 W h Kg^−1^) and 3.75 times higher power density (37.5 KW Kg^−1^) than a similar morphology of few-layer graphene-like nanosheets achieved from waste coconut shell (9.58 W h Kg^−1^ at a maximum power delivery of 10 KW Kg^−1^)^12^.

**Table 2:** Comparison of capacitive performances for various carbon materials synthesized from waste biomass.

Sl. No.	Waste Source	Product morphology	BET Surface area (m^2^/g)	Electrolyte	Potential window	Energy density (W h/ kg)	Power density (kW/Kg)	References
1	Coconut Shell	Sheet-like graphitic carbon	1874	6 M KOH	−1.1-(−0.2 V)	9.58	10	12
2	Wood sawdust	Activated carbon fiber	2294	6 M KOH	0–1 V	7.8	5	78
3	Fish scale	Hierarchical lamellar porous carbon	2273	—	0–1 V	—	—	74
4	Dried Distillers Grains	Porous activated carbon	1021	6 M KOH	−1–0 V	30.14	2.5	75
5	Waste coffee beans	Nanoporous carbon	1840	1 M H_2_SO_4_	0–1 V	20	6	76
6	Sugarcane bagasse	Activated carbon	1788	1 M H_2_SO_4_	0–1 V	10	10	77
7	Rice husk	Microporous carbon	1442	6 M KOH	−0.5–0.5 V	8.36	—	48
8	Dead neem leaves	Microporous carbon	1230	1 M H_2_SO_4_	0–1 V	55.5	11.685	21
9	Peanut shell	Mesoporous carbon	1527	6 M KOH	−0.5–0.5 V	6.68	—	46
10	**Peanut shell**	**Few-layer graphene**	**2070**	**1 M H**_**2**_ **SO**_**4**_	**−0.4–1.1 V**	**58.25**	**37.5**	**This work**

### Performance evaluation of the fabricated symmetric solid-state supercapacitor

In order to ascertain the commercial application and performance of the developed PS-FLG material, we fabricated a symmetric solid-state device (SSD) by coating the synthesized PS-FLG onto a conducting substrate (ITO) and using PVA-H_2_SO_4_ as the gel electrolyte (Fig. 8c, inset and Fig. S11). Microscopic SEM images of the solid-state electrode surface are displayed in Fig. S12, showing the abundance of sheet-structures at low magnification. First, the performance and output deliverance of SSD was characterized and the voltage behavior was monitored using a multimeter (Supporting video, Fig. S13). The discharge voltage with respect to time was monitored as described in Fig. 8c. After charging the device using a constant DC power supply (3 V) for 60 s, the discharge voltage was 1.15 V and monitored for 180 s thereafter. The discharge voltage was noted as 0.70 V, 0.55 V, 0.44 V and 0.41 V after 30 s, 60 s, 120 s, and 180 s, respectively. The voltage retention is around 0.4 V more than 240 s. To further demonstrate the practical application of the device, two devices (PS-FLG/PVA-H_2_SO_4_/ITO) were connected in series. The connected devices were charged up to 3 Volts using a constant DC power supply and were then disconnected. The devices in series were able to light a commercial red LED (1.5 V) successfully (Fig. 8d). These results infer that the developed PS-FLG as electrode material exhibits significant possibilities in energy application.

## Conclusion

In summary, we have developed a new approach for the synthesis of few-layered graphene from no-value biomass waste peanut shell without using any graphitizing agents. The PS-FLG materials possess remarkably high specific surface area and satisfactorily large pore volume. The adsorption-desorption isotherm of PS-FLG reveals that the materials have both the micropores and mesopores. The PS-FLG material is suitable for application in supercapacitor and shows high specific capacitance of 186 F g^−1^ without using any binder in 1 M H_2_SO_4_ as supporting electrolyte. The PS-FLG exhibits highest energy density of 58.13 W h Kg^−1^ and highest power density of 37.5 KW Kg^−1^ in an aqueous electrolyte of 1 M H_2_SO_4_. The specific capacitance and energy density of the PS-FLG material was also tested in an organic electrolyte (1 M Et_4_NBF_4_ in 1:1 mixture of EC: DCE). The specific capacitance of 78.3 F g^−1^ at a current density of 1 A g^−1^ and a sufficiently high energy density of 68 W h Kg^−1^ was observed. The high specific capacitance and energy density can be explained by the presence of micropores with mesopores and high surface area of the PS-FLG materials. We have also synthesized walnut shell and almond shell derived carbon for comparison purpose, and the PS-FLG possesses approximately four times more specific capacitance than WS-C and two times more than AS-C. A solid-state supercapacitor device was fabricated with the PS-FLG materials with ITO as a current collector to demonstrate the possible application and performance of the material. We have shown that the PS-FLG materials have the potential for application as supercapacitor device.

## Electronic supplementary material

Supplementary Information

Supplementary Video
