# Supplementary material for: Large area few-layer graphene with scalable preparation from waste biomass for high-performance supercapacitor
Source: Sci Rep. 2017 Nov 10;7:15239. doi: 10.1038/s41598-017-15463-w (PMC5681691; doi:10.1038/s41598-017-15463-w)
Supplement: Supplementary file 1 — Supplementary Information [file 41598_2017_15463_MOESM1_ESM.pdf]

Supporting Information

Large area few-layered graphene with scalable preparation from waste biomass for high performance supercapacitor

Taniya Purkait, Guneet Singh, Mandeep Singh, Dinesh Kumar, Ramendra Sundar Dey*

Institute of Nano Science and Technology (INST), Mohali-160064, Punjab, India.
Email: rsdey@inst.ac.in (R. S. Dey)

Formula and equation used in the manuscript.

The specific capacitance (C_{sp}) can be calculated from cyclic voltammetry via the equation

$$C_{sp} = \frac{I_{avg}}{\mathrm{m}\Delta \nu} \qquad (1)$$

where I_{avg} is the average current obtained from cathodic and anodic sweeps, υ is the scan rate, and m is the active mass of the pErGO material interdigitated onto the electrode surface.

The specific capacitance, C_{SP} was also calculated from galvanostatic charge-discharge curves by equation (2) as following

$$C_{SP} = \frac{I\Delta t}{\Delta V}\frac{1}{M} \qquad (2)$$

where, I (in A) is the discharge current, Δt (in s) is the discharge time, M (in g) is the active mass of device, and ΔV (in V) is the working voltage.

The energy density (E) and power density (P) of the supercapacitor system can be expressed by equation. (3) and (4) as following

$$E = \frac{\Delta V^2}{2\times 3600} C_{SP} \qquad (3)$$

$$P = \frac{E\times 3600}{\Delta t} \qquad (4)$$

Where (C_{sp}) is the mass specific capacitance.

Supporting data

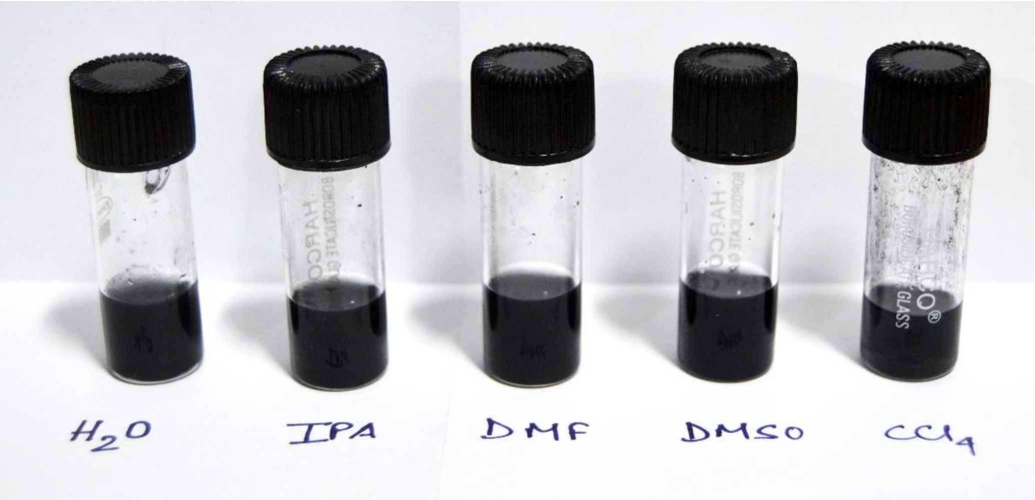

Figure S1: Digital picture of PS-FLG dispersed in solvent of different polarities like water, isopropanol, dimethylformamide, dimethyl sulfoxide and carbon tetrachloride.

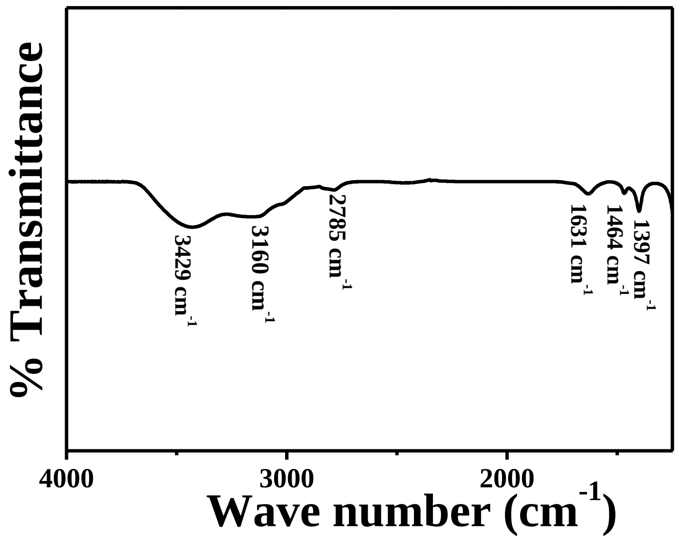

Figure S2: FT-IR spectrum of PS-FLG

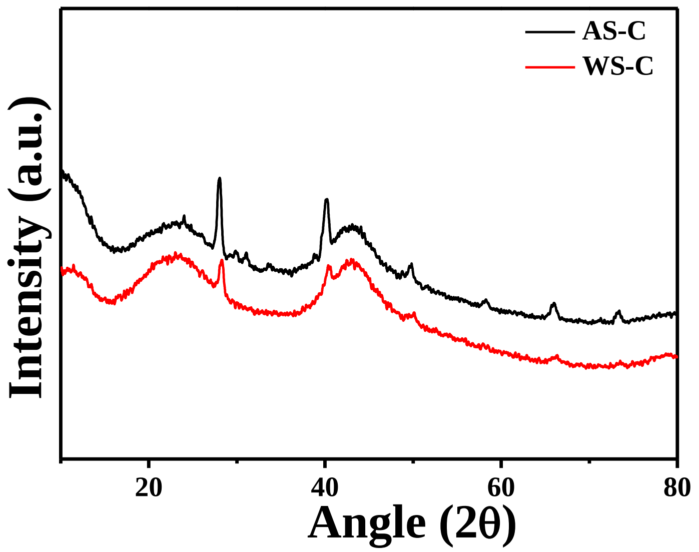

Figure S3: Comparative XRD analysis of WS-C and AS-C.

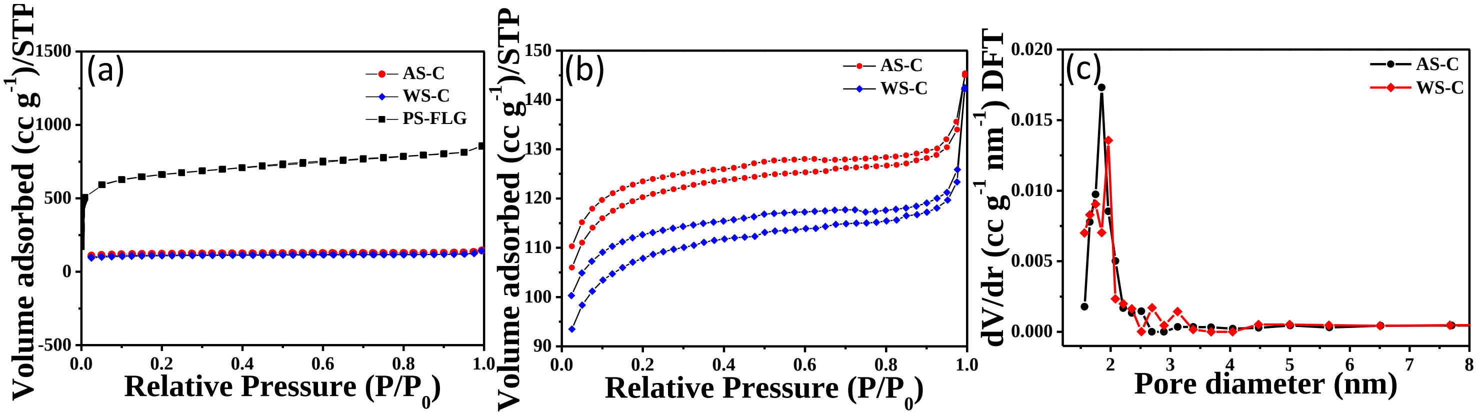

Figure S4: (a) Comparison of N_2 sorption analysis isotherm of PS-FLG with other nutshell derived carbon and (b) Magnified view of figure (a): BET adsorption isotherm plots of AS-C and WS-C. (c) pore size distribution plots of AS-C and WS-C.

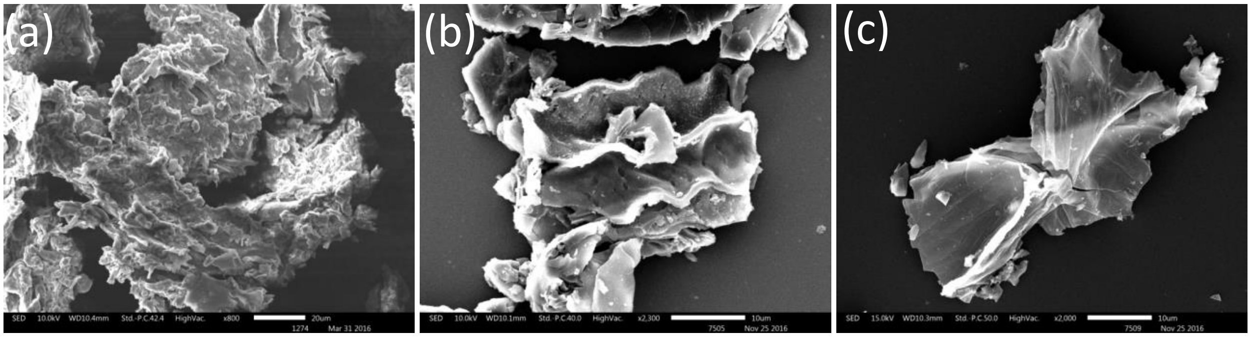

Figure S5. Microscopic surface analysis: SEM images of (a) Pre-carbonized sample (PS-P), (b) PS-C, (c) PS-G. Scale Bars: (a) 20 µm, (b) 10 µm & (c) 10 µm respectively.

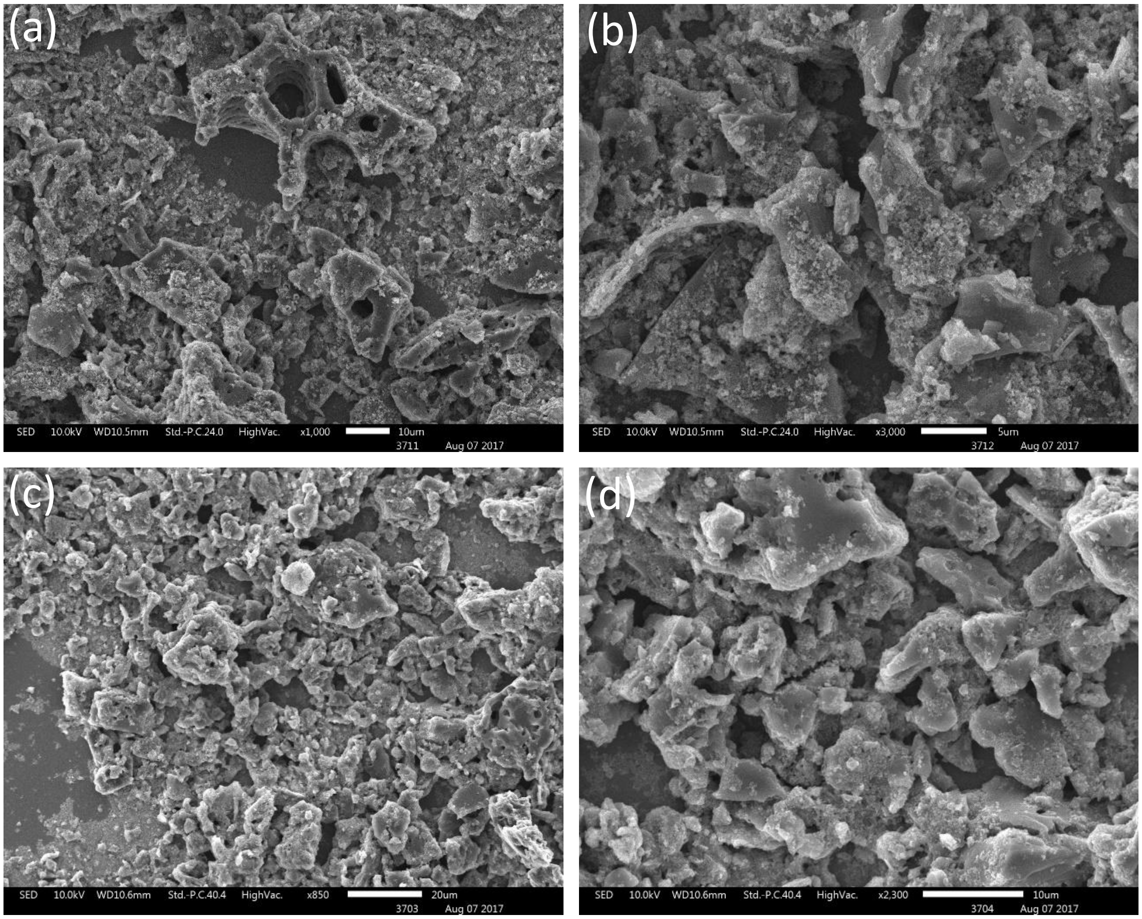

Figure S6. Comparative surface morphological studies: (a) & (b) Microscopic SEM images of Almond Shell Carbon (AS-C). Scale bar: 10 µm & 5 µm. (c) & (d) SEM images of Walnut Shell Carbon (WS-C). Scale bar: 20 µm & 10 µm.

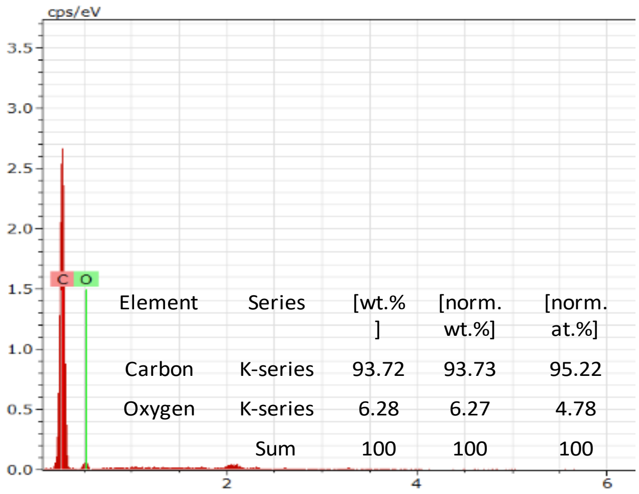

Element	Series	[wt.%]	[norm. wt.%]	[norm. at.%]
Carbon	K-series	93.72	93.73	95.22
Oxygen	K-series	6.28	6.27	4.78
	Sum	100	100	100

Figure S7. EDS graphs showing elemental distributions in PS-FLG.

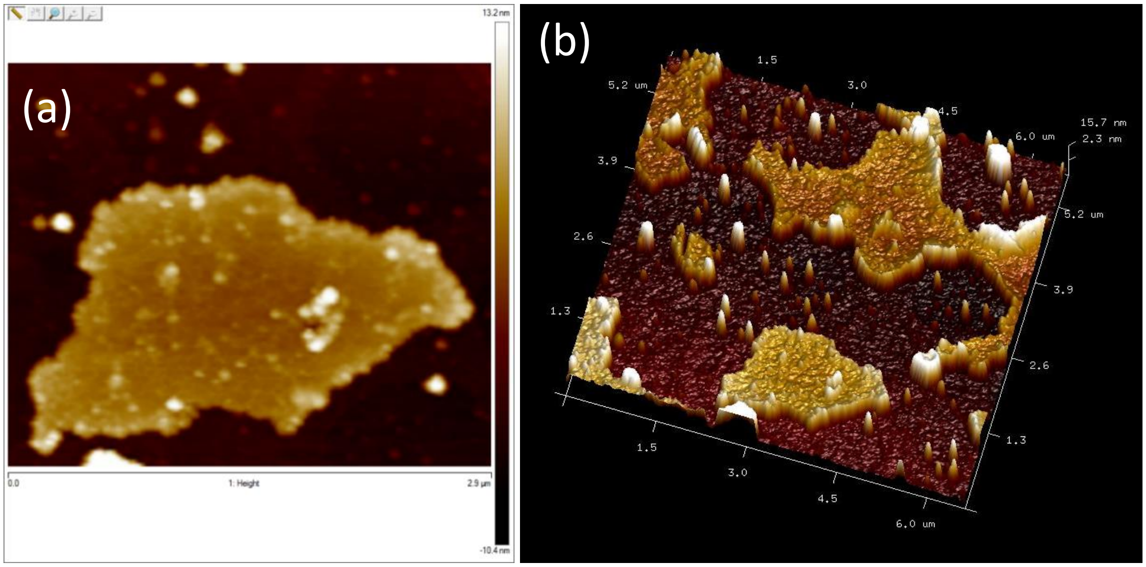

Figure S8. Tapping-mode AFM images of PS-FLG: (a) 2D image at 2.9 µm (b) 3D mapping of PS-FLG.

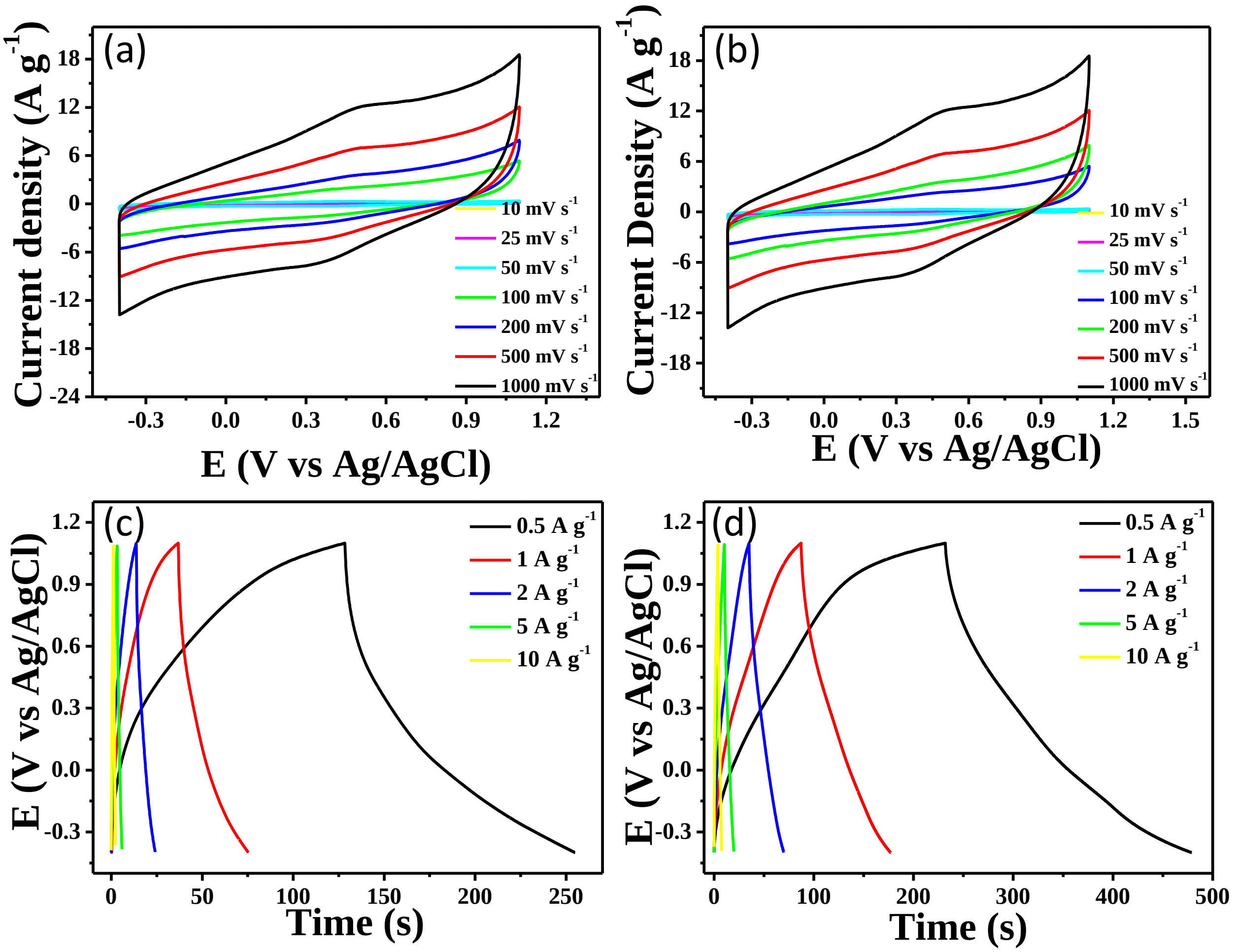

Figure S9: Cyclic voltammogram of (a) WS-C and (b) AS-C at different scan rates. Galvanostatic charge-discharge profile of (c) WS-C and (d) AS-C at different current densities.

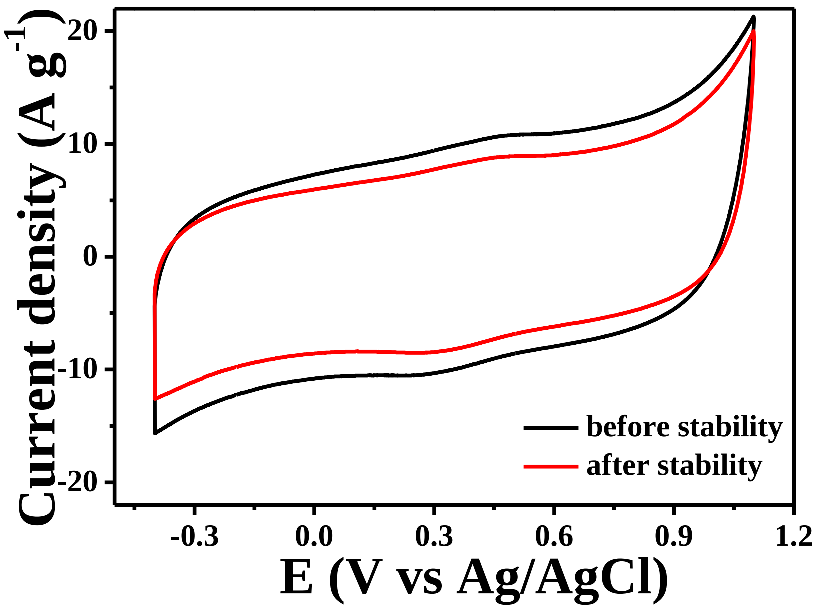

Figure S10: CV curves obtained before and after 5000 cycles of GCD at 10 A g^{-1}

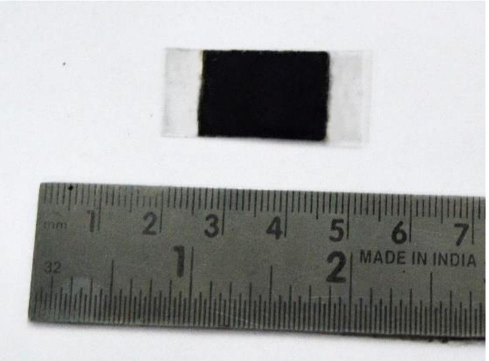

Figure S11: Digital picture of PS-FLG based solid-state supercapacitor device.

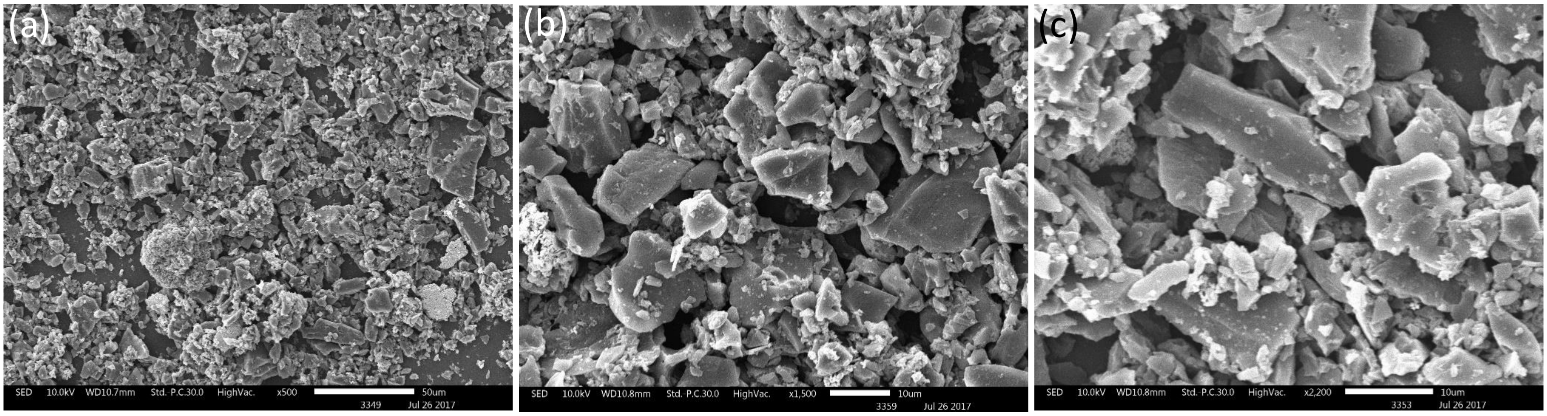

Figure S12. Microscopic SEM images of the solid-state device surface after modifying with the active material, PS-FLG. Scale bar: 50 μm, 10 μm & 10 μm.

Figure S13: Supporting video describes the performance and output deliverance of a supercapacitor device after charging it for 60 sec. the discharging performance of the device was monitored for 180 s.

File name: Supporting video-1

(See video attachment for details)

Table S1: Summarization of surface parameters of PS based materials

**Samples**	**S_{BET} (m^2g^{-1})**	**Pore size (nm)**	**Pore Volume (cc g^{-1})**
PS-C	645	1.14	0.37
PS-G	1554	1.22	0.93
**PS-FLG**	**2070**	**1.28**	**1.33**
WS-C	363	1.85	0.047
AS-C	403	1.96	0.035
